# Dead tired: evaluating the physiological status and survival of neonatal reef sharks under stress

**DOI:** 10.1093/conphys/coy053

**Published:** 2018-09-18

**Authors:** Ian A Bouyoucos, Ornella C Weideli, Serge Planes, Colin A Simpfendorfer, Jodie L Rummer

**Affiliations:** 1 Australian Research Council Centre of Excellence for Coral Reef Studies, James Cook University, Townsville, Queensland, Australia; 2 PSL Research University, EPHE-UPVD-CNRS, USR 3278 CRIOBE, Université de Perpignan, 58 Avenue Paul Alduy, Perpignan Cedex, France; 3 Laboratoire d’Excellence “CORAIL”, EPHE, PSL Research University, UPVD, CNRS, USR 3278 CRIOBE, Papetoai, Moorea, French Polynesia; 4 Centre for Sustainable Tropical Fisheries and Aquaculture & College of Science and Engineering, James Cook University, Townsville, Queensland, Australia

**Keywords:** Bycatch, marine protected areas, oxygen uptake rates, physiological stress response, shark nursery areas, temperature

## Abstract

Marine protected areas (MPAs) can protect shark populations from targeted fisheries, but resident shark populations may remain exposed to stressors like capture as bycatch and environmental change. Populations of young sharks that rely on shallow coastal habitats, e.g. as nursery areas, may be at risk of experiencing these stressors. The purpose of this study was to characterize various components of the physiological stress response of neonatal reef sharks following exposure to an exhaustive challenge under relevant environmental conditions. To accomplish this, we monitored markers of the secondary stress response and measured oxygen uptake rates ($\dot{M}O_{2}$) to compare to laboratory-derived baseline values in neonatal blacktip reef (*Carcharhinus melanopterus*) and sicklefin lemon sharks (*Negaprion acutidens*). Measurements occurred over three hours following exposure to an exhaustive challenge (gill-net capture with air exposure). Blood lactate concentrations and pH deviated from baseline values at the 3-h sample, indicating that both species were still stressed 3 h after capture. Evidence of a temperature effect on physiological status of either species was equivocal over 28–31°C. However, aspects of the physiological response were species-specific; *N. acutidens* exhibited a larger difference in blood pH relative to baseline values than *C. melanopterus*, possibly owing to higher minimum $\dot{M}O_{2}$. Neither species experienced immediate mortality during the exhaustive challenge; although, single instances of delayed mortality were documented for each species. Energetic costs and recovery times could be extrapolated for *C. melanopterus via* respirometry; sharks were estimated to expend 9.9 kJ kg^−1^ (15% of energy expended on daily swimming) for a single challenge and could require 8.4 h to recover. These data suggest that neonatal *C. melanopterus* and *N. acutidens* are resilient to brief gill-net capture durations, but this was under a narrow temperature range. Defining species’ vulnerability to stressors is important for understanding the efficacy of shark conservation tools, including MPAs.

## Teaser

Marine protected areas can protect sharks from targeted fisheries, but may not manage other stressors. We demonstrated species-specific physiological responses of newborn reef sharks upon brief gill-net capture during summer months. *Carcharhinus melanopterus* and *Negaprion acutidens* were resilient to stress within a narrow temperature range and under ideal capture conditions.

## Introduction

Marine protected areas (MPA), including shark sanctuaries, can be important conservation tools for protecting threatened shark populations. Indeed, some shark populations face declines worldwide, owing to overexploitation in fisheries (Dulvy *et al.*, 2014). One strategy to potentially reduce the threat of fishing to shark populations is through the creation of MPAs with specific regulations that protect shark populations. For instance, “shark sanctuaries” ban targeted shark fisheries within a country’s exclusive economic zone (EEZ) (Cramp *et al.*, 2018). A general concern regarding protected habitats for sharks and other top predators is that other significant threats, like bycatch or environmental change, are not adequately managed (Ward-Paige and Worm, 2017). Incidental capture, or bycatch, affects shark populations through fishing-induced mortality and negative sub-lethal outcomes (Skomal and Mandelman, 2012; Wilson *et al.*, 2014; Ellis *et al.*, 2017). Climate change is resulting in ocean warming and acidification and can affect shark populations through local extirpation as conditions become too extreme in addition to negative sub-lethal outcomes (Rosa *et al.*, 2017; Payne *et al.*, 2018). Protected shark populations may be inherently at risk of experiencing negative outcomes associated with these stressors because virtually all shark sanctuaries are in the tropics. Here, environmental conditions may border species’ limits to optimal physiological performance and therefore impede resolving stressors (Rummer *et al.*, 2014). Furthermore, populations that rely on shallow coastal waters during key parts of their life histories (e.g. neonates in nursery areas) are already facing quite variable environmental conditions and can also be risk of fishing interactions (Knip *et al.*, 2010). Therefore, developing an understanding of shark populations’ resilience to stressors that they are still expected to face within MPAs can provide valuable information for improving the efficacy of these conservation tools (Chin *et al.*, 2010; Illing and Rummer, 2017).

Neonatal and juvenile shark populations that rely on nearshore habitats may be vulnerable to bycatch. Shallow coastal environments are important for young sharks as nursery areas (Heupel *et al.*, 2007). Alternatively, non-nursery areas can provide stability to young shark populations that typically utilize a diversity of habitats (Yates *et al.*, 2012). While shallow waters may offer young sharks protection from predators, proximity to the coastline increases the probability of fishing interactions (Knip *et al.*, 2010). Specifically, young sharks can be caught as bycatch in artisanal and recreational fisheries. Depending on the type of fishery (e.g. hook-and-line or net fishing), different species have varying susceptibilities to lethal or sub-lethal outcomes (Dapp *et al.*, 2016). Capture is generally associated with vigorous escape attempts that can drive a physiological stress response (Brooks *et al.*, 2012; Guida *et al.*, 2016; Gallagher *et al.*, 2017). The stress response is generally characterized by a release of hormones (e.g. adrenaline and noradrenaline), the accumulation of by-products of anaerobic metabolism (e.g. lactate) that drive declines in tissue pH, and resultant osmotic and ion imbalances (Skomal and Mandelman, 2012). Capture is also associated with an increased rate of energy expenditure (Bouyoucos *et al.*, 2017b). Physiological stress and depleted energy reserves following fisheries capture can even contribute to exhaustion-induced mortality or post-release predation (Danylchuk *et al.*, 2014; Lennox *et al.*, 2017). Additional lethal stressors can be problematic because young sharks may already experience high mortality rates during their first year of life (Gruber *et al.*, 2001; Heupel and Simpfendorfer, 2002).

Young shark populations in shallow coastal habitats must also contend with stressors associated with variable environmental conditions. Shallow coastal environments can be prone to seasonal and tidal variations in environmental conditions, such as temperature, salinity and dissolved oxygen concentrations that affect the abundance and distribution of various species of sharks (Knip *et al.*, 2010; Schlaff *et al.*, 2014; Oh *et al.*, 2017b). Changes in abundance and distribution may be partially attributed to physiological costs associated with variable environmental conditions. Increases in temperature decrease oxygen’s solubility in water. Oxygen uptake rates (a proxy for metabolic rate) also increase, along with concomitant decreases in haemoglobin–oxygen (Hb–O_2_) affinity (Bernal *et al.*, 2012, 2018). In addition, parameters associated with sharks’ stress response to capture vary with temperature, such that capture at high temperatures can be fatal for some species (Hoffmayer *et al.*, 2012; Danylchuk *et al.*, 2014; Guida *et al.*, 2016). While sharks may attempt to maintain a preferred body temperature or boundaries to their critical thermal limits, life history stages (e.g. neonates) that derive specific benefits from confined habitats (e.g. predator avoidance within nursery areas) must be able to tolerate local conditions (Knip *et al.*, 2010; Payne *et al.*, 2016, 2018). However, sharks in the tropics are expected to be adapted to a narrow range of temperatures and, therefore, to have a low tolerance for variable environmental temperature conditions (Rummer and Munday, 2017). While there is a paucity of data on thermal tolerance limits for sharks, it is likely that sharks within coastal habitats in tropical latitudes may already be living close to their thermal tolerance limits (Rummer *et al.*, 2014).

The purpose of this study was to characterize various components of the stress response of neonatal reef sharks following an exhaustive challenge. Specifically, we sought to measure the physiological status of neonatal blacktip reef sharks (*Carcharhinus melanopterus*) and sicklefin lemon sharks (*Negaprion acutidens*) at multiple points in time following *in situ* gill-net capture. The objectives of this study were to (1) characterize physiological responses in neonatal reef sharks following capture, (2) predict the effect of changes in environmental temperatures on physiological status, (3) assess the differential vulnerability of co-occurring neonatal reef shark species to stress-induced physiological impairment and (4) estimate the energetic cost of an exhaustive challenge in the context of routine energy requirements. Studies of this nature are necessary for understanding whether stressors hold lethal or sub-lethal consequences under predictable environmental conditions in important habitats like shark nursery areas. As such, these data will have management applications to better support conservation initiatives for reef sharks (Illing and Rummer, 2017).

## Materials and methods

All experiments were approved by James Cook University Animal Ethics Committee protocol A2089. Research on sharks in French Polynesia was approved under Arrêté N° 9524 issued by the Ministère de la Promotion des Langues, de la Culture, de la Communication et de l’Environnement of the French Polynesian government on 30 October 2015.

### Study site, animal collection and husbandry

Fieldwork was conducted from shore around Moorea, French Polynesia (17°30′S, 149°51′W), where targeted shark fishing in the country’s EEZ has been banned since 2012 (Ward-Paige and Worm, 2017). Newborn *C. melanopterus* and *N. acutidens* are abundant during parturition months from September through February (Mourier and Planes, 2013; Mourier *et al.*, 2013a, b). Sharks were collected during November and December 2016 using monofilament gill-nets (50.0 m × 1.5 m, 5.0 cm mesh) fished at dusk (17:00–20:00). Captured sharks were immediately identified and removed from the net in under five minutes. Prior to release, biological data (total length, mass, and sex) were collected from all sharks. Individuals were tagged with coloured T-bar anchor tags (Hallprint, Hindmarsh Valley, SA, Australia) to avoid repeatedly sampling recaptured animals for this study. Only animals in good condition (e.g. without open or healing bite wounds or retained fish hooks) were sampled for this study. Environmental temperatures were recorded every ten minutes with one or two temperature data loggers (UA-002-64, Onset Computer Corporation, Bourne, MA, USA) that were deployed in a transect parallel to the gill-net.

A subset of sharks was transported to the Centre de Recherches Insulaires et Observatoire de l’Environnement (CRIOBE) by vehicle in 200.0 l insulated coolers of aerated seawater (Chin *et al.*, 2015). Before transport, sharks were retained in individual flow-through mesh bags (0.2 m diameter and 1.0 m long) for no more than 1 h prior to transport. Including transport, sharks were typically confined for under 90 min post-capture before arriving at the CRIOBE’s holding facility. Sharks were housed in 1250 l circular flow-through tanks (2–3 sharks per tank), and *C. melanopterus* and *N. acutidens* were separated. Tanks were covered with 60% shade cloth, continuously aerated and supplied filtered seawater from an offshore pump. The holding facility was covered and open-sided, exposing sharks to a natural photoperiod. Sharks were held for at least one week before experimentation and were fed 5.0% of their body mass in fresh tuna every other day (Chin *et al.*, 2015) with the exception of a 48-h fasting period prior to their use in experiments. All sharks were released to their original capture site after no more than 4 weeks in captivity.

### Quantifying physiological responses

Physiological responses to an exhaustive challenge were quantified for *C. melanopterus* and *N. acutidens*. The exhaustive challenge used throughout the entire study included approximately ~3 min of gill-net capture (3.4 ± 1.2 min SD) and 1 min of air exposure. Gill-net capture has been demonstrated to induce exhaustion in elasmobranchs (Frick *et al.*, 2009, 2010, 2012)—including juvenile *C. melanopterus* and *N. acutidens* (Dapp *et al.*, 2017)—and a standardized duration of air exposure is commonly employed along with an exhaustive challenge to maximally exhaust fish and to simulate handling of fish out of water by fishers (Clark *et al.*, 2013; Rummer *et al.*, 2016). Values for physiological metrics were generated from unique individuals subjected to one of four treatments. One group of laboratory-acclimated sharks was phlebotomized in a quiescent state after 2–4 weeks in captivity and a 48-hour fasting period to generate minimally-stressed values (“baseline” treatment). A second group of sharks was phlebotomized immediately following the exhaustive challenge in the field (“immediate” treatment). The third group of sharks faced the same exhaustive challenge and was retained in flow-through mesh bags in the field for 3 h before phlebotomy (“three-hour” treatment). A final group of sharks was sampled after 3 h in a respirometry chamber that was used to estimate energetic costs and recovery times for the exhaustive challenge (“respirometry” treatment). All blood samples were processed immediately following phlebotomy.

Sharks were phlebotomized via caudal puncture using heparin-rinsed 23.0 gauge 3.8 cm needles. Five parameters were measured using point-of-care analytical devices: blood glucose concentration (mmol l^−1^), blood lactate concentration (mmol l^−1^), blood pH, haemoglobin concentration ([Hb]; g dl^−1^), and haematocrit (Hct). Blood was first transferred from syringes directly to two 70-μl microcapillary tubes that were run in parallel in a microhaematocrit centrifuge (ZIPocrit, LW Scientific, Lawrenceville, GA, USA) for 2 min at 4400 *g* (Danylchuk *et al.*, 2014). Whole blood glucose and lactate concentrations were measured with 10 μl samples of whole blood using an Accutrend Plus (Roche Diagnostics Ltd, Rotkreuz, Switzerland), with ranges of 1.1–33.3 mmol l^−1^ and 0.8–22.0 mmol l^−1^, respectively (Butcher *et al.*, 2015). Readings that were outside the measurement range were reported as the value of the upper or lower device limit for statistical analyses. Haemoglobin concentration was measured with a HemoCue Hb 201 System (Australia Pty Ltd, Victoria, Australia) using 10 μl of whole blood, and was corrected using a calibration equation generated for fish that has previously been applied to sharks (Clark *et al.*, 2008; Heinrich *et al.*, 2014). Haemoglobin concentration was then converted to tetramer Hb concentration (Hb_4_, in mmol l^−1^) using conversions generated for tropical reef species in order to calculate mean cell haemoglobin concentration (MCHC; mmol l^−1^), as Hb_4_ divided by Hct (Rummer *et al.*, 2013; Heinrich *et al.*, 2014). Blood pH was measured using a HI98165 pH meter (Hanna Instruments, Victoria, Australia), and raw pH values were converted to values derived from the conventional i-STAT system using a correction formula generated for juvenile lemon sharks (*N. brevirostris*) at 25.6–31.3°C (Talwar *et al.*, 2017).

### Estimating energetic costs and recovery

To estimate costs of an exhaustive challenge and recovery times, individuals from another subset of sharks (*C. melanopterus*) were, transferred to individual field respirometry chambers immediately after capture and air exposure so that oxygen uptake rates ($\dot{M}O_{2}$, in mg O_2_ kg^−1^ h^−1^) could be measured over 3 h. To do this, two respirometry chambers (24.0 cm diameter and 70.0 cm long, 32.0 l volume including tubing) were submerged in a 400.0 l circular pool positioned ~3.0 m from the shoreline. Water in the pool was continuously aerated, and was supplied at a rate of 4800.0 l h^−1^ from a pump approximately 5.0 m offshore in at least 0.3 m of water. Respirometry chambers were configured for intermittent-flow respirometry with 2500.0 l h^−1^ flush and recirculating pumps (Rummer *et al.*, 2016; Svendsen *et al.*, 2016). Dissolved oxygen concentration (DO, in mg l^−1^) was measured every second with fibre optic probes that were mounted within chambers and connected to a Firesting Optical Oxygen Meter (Pyroscience, Aachen, Germany). Probes were calibrated to fully-aerated freshwater (100.0% saturation) before each use and to 0.0% saturation with sodium sulphite as needed. Flush pumps were manually operated to cycle flush (9.1 ± 6.5 min SD) and measurement periods (11.5 ± 6.1 min) such that DO remained above 80.0% air saturation. The timing of cycles was determined by watching DO in real-time on a laptop computer. Sharks were placed into the chambers immediately upon capture, and therefore the time from the onset of capture to the beginning of the first measurement was 4.4 ± 1.2 min (i.e. the length of the exhaustive challenge). Each field respirometry trial consisted of 6–12 measurement periods over 3 h. Then, immediately after removal from respirometry chambers, all sharks were phlebotomized to determine whether undergoing respirometry influenced the stress response (the “respirometry” treatment).

Oxygen uptake rates were estimated by first calculating rates of DO decline every 30 s during each measurement using LabChart (7.3.8, ADInstruments, Dunedin, New Zealand). Specifically, $\dot{M}O_{2}$ was calculated as ${\dot{M}O}_{2}\;=\;SV_{\mathrm{Resp}}M^{-1}$ where *S* is the slope of the linear decline in DO (in mg O_2_ L^−1^ s^−1^), *V*_Resp_ is the volume of the respirometer minus the shark’s volume (in l), and *M* is the mass of the fish (in kg). Background respiration was accounted for by modelling the linear increase in background $\dot{M}O_{2}$, measured before and after each trial in chambers without fish, and subtracting proportional background $\dot{M}O_{2}$ from each $\dot{M}O_{2}$ measurement (Rodgers *et al.*, 2016; Rummer *et al.*, 2016). The highest $\dot{M}O_{2}$ during each measurement period was selected, and these values were fit with an exponential decay curve (recovery curve). The highest $\dot{M}O_{2}$ value for each shark was recorded as its maximum $\dot{M}O_{2}$ ($\dot{M}O_{2{}_{\mathrm{Max}}}$).

Oxygen uptake rates of minimally-stressed, resting sharks (*C. melanopterus* and *N. acutidens*; the same animals used in for the “baseline” treatment) were measured in the laboratory. The same respirometry chambers described above were placed in holding tanks, and flush pumps were automated with a custom-built data acquisition system and software (National Instruments, Austin, Texas, USA). Flush pumps were automated to shut off for 5 min every 12 min for *C. melanopterus*, and 5 min every 15 min for *N. acutidens*, yielding at least 120 measurements for *C. melanopterus* and at least 96 measurements for *N. acutidens* over 24 h. Shorter measurement periods and longer flush periods were deemed necessary for *N. acutidens* because all individuals were larger than the *C. melanopterus* used for this study and had higher $\dot{M}O_{2}$. One slope (*S*) was calculated for each measurement. Minimum ($\dot{M}O_{2{}_{\mathrm{Min}}}$) was calculated as the mean of the lowest 10% of $\dot{M}O_{2}$ values, excluding values outside of the mean ± 2 SD (Clark *et al.*, 2013).

### Statistical and data analyses

Underlying physiological responses were characterized by comparing values of physiological parameters over time after an exhaustive challenge, and against baseline values. The influence of temperature on physiological status (i.e. values of physiological and oxygen uptake parameters) was assessed by including temperature as a covariate in models. Physiological parameters (i.e. blood glucose and lactate concentrations, blood pH, [Hb], Hct and MCHC) were fit with linear models to observe variation in responses with treatment (fixed effect), temperature and mass (covariates) for both species. For *C. melanopterus*, the factor “treatment” had four levels (i.e. baseline, immediate, three-hour, and respirometry). It was not possible to catch comparable numbers of *N. acutidens*, and as a result the factor “treatment” only had three levels (i.e. baseline, immediate and 3-h). All possible interactions (two-way and three-way) were included in these models for *C. melanopterus*. Samples sizes were too small to include interactions for *N. acutidens*. Post hoc multiple comparisons were made with Tukey’s honest significant difference (HSD) tests. Models were validated with Q–Q plots of model residuals, and by plotting residuals against treatment and fitted values (Zuur *et al.*, 2007). For all tests, the acceptable Type I error rate (α) was 0.05, and all analyses were conducted using the R Stats Package (R Core Team, 2016).

Recovery times and costs were estimated for *C. melanopterus* using respirometry data. The mean value of *Ṁ*O_2Min_ that was derived from the laboratory was used as a baseline for estimating the excess post-exercise oxygen consumption (EPOC, in mg O_2_ kg^−1^) of individual sharks from field respirometry. Recovery times were estimated for individual sharks as the time when the recovery curve intersected the upper 95% confidence interval limit of *Ṁ*O_2Min_ (Bouyoucos *et al.*, 2017a). Excess post-exercise oxygen consumption, which represents the cost of recovery from exhaustive activity (Gaesser and Brooks, 1984), was calculated as the area bound by individual sharks’ recovery curves, *Ṁ*O_2Min_, the time of the first *Ṁ*O_2_ measurement, and the time of recovery (Bouyoucos *et al.*, 2017a). Oxygen uptake parameters (i.e. *Ṁ*O_2Min_, *Ṁ*O_2Max_, EPOC and recovery time) were fit with linear models to observe variation with temperature and mass, including interactions.

## Results

### Quantifying physiological responses

**Figure 1:**
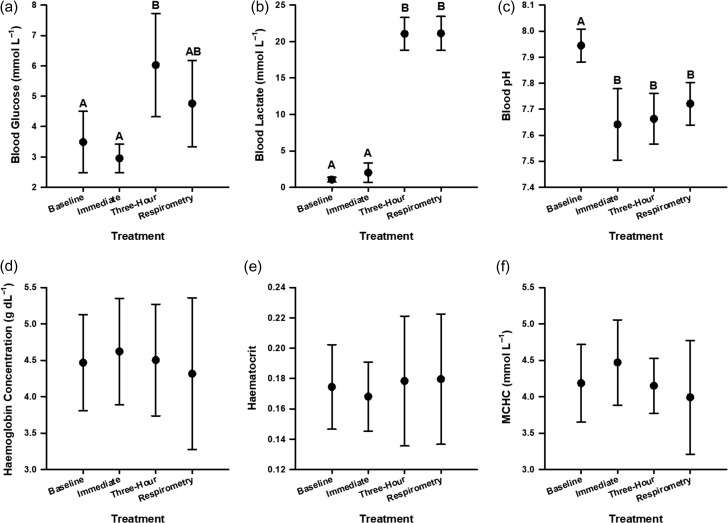
Indicators of the stress response in juvenile blacktip reef sharks (*Carcharhinus melanopterus*) following an exhaustive challenge *in situ*. Baseline values were taken from quiescent, fasted sharks (“baseline”). Other sharks were phlebotomized immediately following exhaustive gill-net capture (“immediate”), after three hours in a recovery bag (“three-hour”) or after 3 h in a field respirometry chamber (“respirometry”). Differing letters denote statistically significant differences. Abbreviation: mean cell haemoglobin concentration (MCHC).

**Figure 2:**
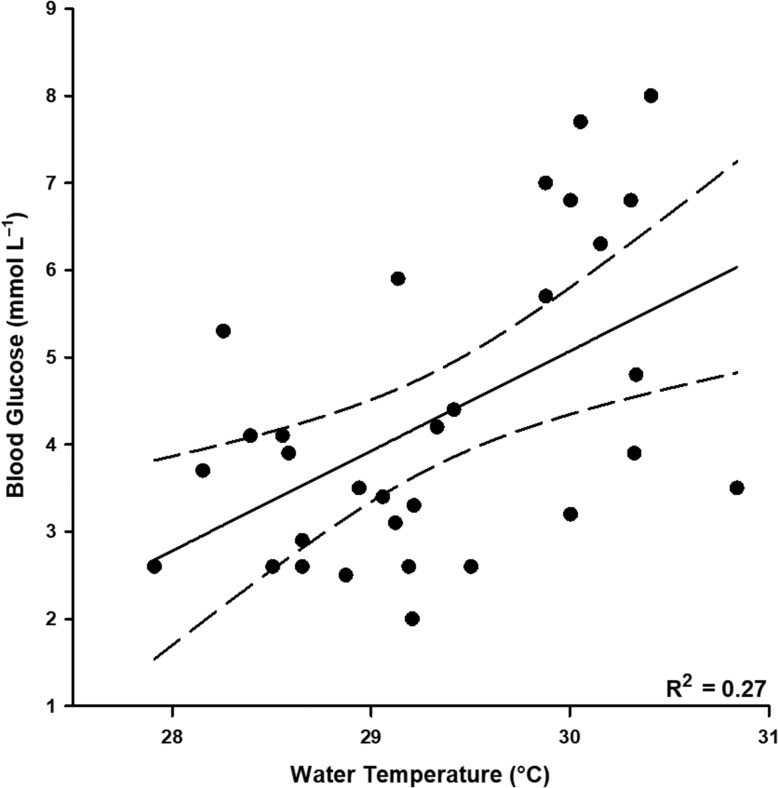
Relationship between temperature and physiological status (blood glucose concentrations) for blacktip reef sharks (*Carcharhinus melanopterus*).

Morphometric data for *C. melanopterus* are presented in Table 1. Sharks exhibited significant changes in blood glucose and lactate concentrations as well as blood pH across treatments (Supplementary Table S1). Blood glucose concentrations at three hours were higher than baseline values (Tukey’s HSD, *t* = 4.387, *P* < 0.001) and values for immediately-sampled sharks (Tukey’s HSD, *t* = 4.062, *P* = 0.002) (Fig. 1a). Blood glucose concentrations also had a positive linear relationship with temperature (Linear regression, *R*^2^ = 0.27, *F*_1, 29_ = 10.82, *P* = 0.003; 27.9–30.9 °C; Fig. 2) across treatments (Supplementary Table S1). Baseline and immediately-sampled values for blood lactate concentrations did not differ (Tukey’s HSD, *t* = 1.436, *P* = 0.489), and values after 3 h in recovery bags and respirometry chambers were not different (Tukey’s HSD, *t* = −0.639, *P* = 0.918). Blood lactate concentrations were at least 14-fold higher 3-h post-capture relative to baseline and immediately-sampled values (Tukey’s HSD, *P* < 0.001) (Fig. 1b). Lastly, blood pH was uniformly reduced across all treatments relative to baseline values (Tukey’s HSD, *P* < 0.001) (Fig. 1c). No significant differences in [Hb] (4.48 ± 0.77 g dl^−1^), Hct (0.17 ± 0.03) or MCHC (4.20 ± 0.58 mmol l^−1^) were detected.

**Table 1:** Morphometric data (mean ± SD), samples sizes by sex and water temperatures by experimental treatment. Baseline values were taken from quiescent, fasted sharks (“baseline”). Other sharks were phlebotomized immediately following exhaustive gill-net capture (“immediate”), after 3 h in a recovery bag (“three-hour”) or after 3 h in a field respirometry chamber (“respirometry”)

Species	Treatment	*n* (f:m)	Total length (mm)	Mass (kg)	Water temperature (°C)
*Carcharhinus melanopterus*	Baseline	6:2	577.88 ± 30.13	1.08 ± 0.16	29.66 ± 0.69
	Immediate	3:5	578.63 ± 31.08	1.08 ± 0.12	28.77 ± 0.49
	3-h	1:7	587.75 ± 32.34	1.18 ± 0.18	29.72 ± 0.83
	Respirometry	3:5	559.13 ± 20.15	1.02 ± 0.12	30.06 ± 1.28
*Negaprion acutidens*	Baseline	1:2	688.67 ± 17.01	1.55 ± 0.26	29.29 ± 0.75
	Immediate	2:6	647.50 ± 35.40	1.45 ± 0.23	30.14 ± 0.49
	3-h	1:3	680.00 ± 5.29	1.49 ± 0.18	29.73 ± 0.25

**Figure 3:**
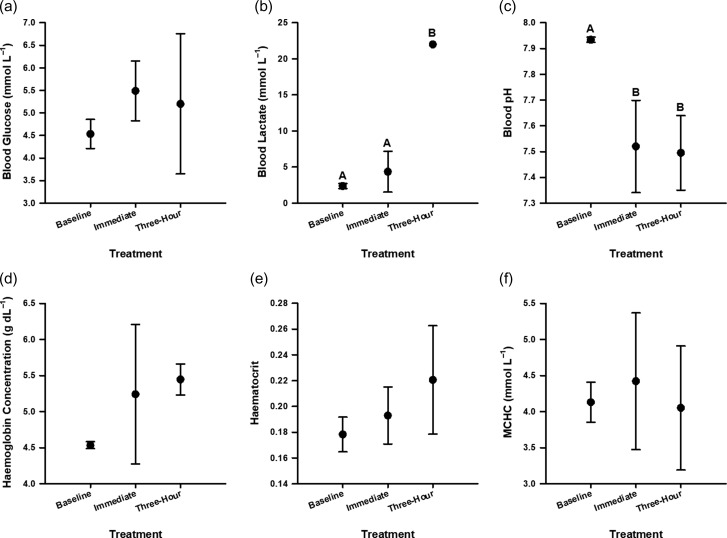
Indicators of the stress response in juvenile sicklefin lemon sharks (*Negaprion acutidens*) following an exhaustive challenge *in situ*. Baseline values were taken from quiescent, fasted sharks (“baseline”). Other sharks were phlebotomized immediately following exhaustive gill-net capture (“immediate”) or after 3 h in a recovery bag (“three-hour”). Differing letters denote statistically significant differences. Abbreviation: mean cell haemoglobin concentration (MCHC).

**Figure 4:**
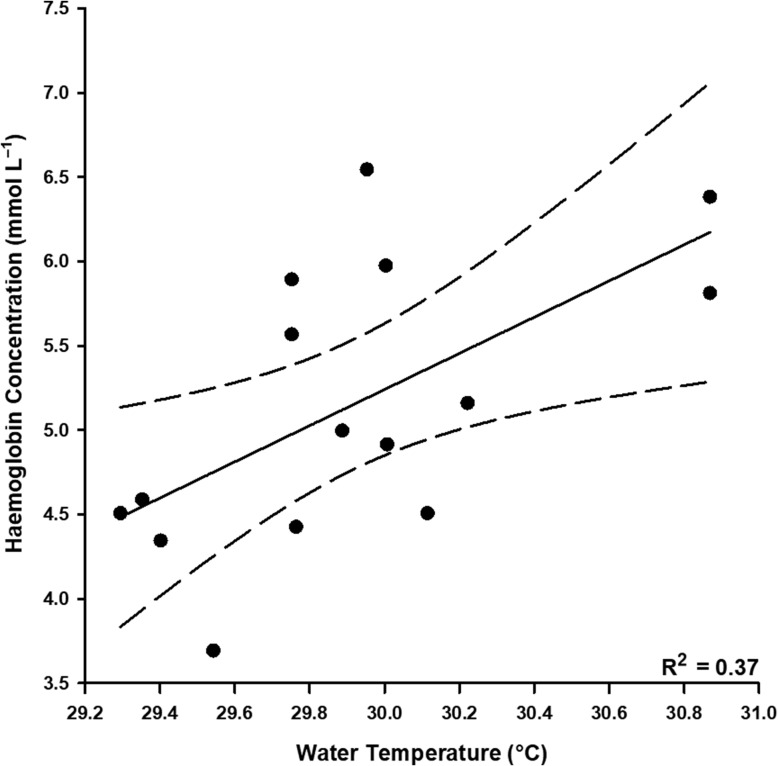
Relationship between temperature and physiological status (haemoglobin concentrations) for sicklefin lemon sharks (*Negaprion acutidens*).

Morphometric data for *N. acutidens* are presented in Table 1. Differences between treatments were only detected for blood lactate concentration and blood pH (Supplementary Table S2). Blood lactate concentrations were at least 6-fold higher for *N. acutidens* three hours after capture relative to baseline (Tukey’s HSD, *t* = 9.128, *P* < 0.001) and immediately-sampled values (Tukey’s HSD, *t* = 8.407, *P* < 0.001), which were not different (Tukey’s HSD, *t* = −1.679, *P* = 0.269) (Fig. 3b). In addition, blood pH was significantly reduced for sharks sampled immediately (Tukey’s HSD, *t* = −3.153, *P* = 0.014) and 3 h post-capture (Tukey’s HSD, *t* = −2.940, *P* = 0.037) relative to baseline pH (Fig. 3c). Blood pH values sampled immediately or three hours post-capture were not different (Tukey’s HSD, *t* = 0.185, *P* = 0.981). There were no significant differences across treatments for blood glucose concentration (5.22 ± 0.88 mmol l^−1^), [Hb] (5.15 ± 0.83 g dl^−1^), Hct (0.19 ± 0.03) or MCHC (4.26 ± 0.77 mmol l^−1^) for *N. acutidens*. No physiological parameter varied with mass (Supplementary Table S2), and [Hb] had a positive linear relationship with temperature (Linear regression, *R*^2^ = 0.37, *F*_1, 13_ = 7.59, *P* = 0.016; 29.5–30.9°C; Fig. 4).

### Estimating energetic costs and recovery

**Figure 5:**
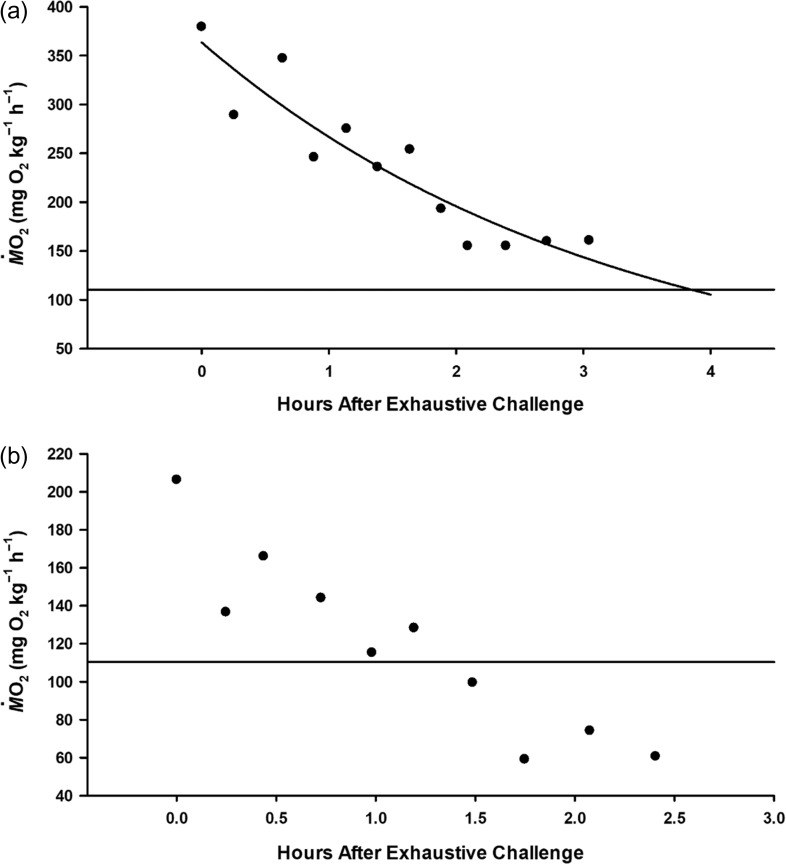
Representative traces of excess post-exercise oxygen consumption (EPOC). Data are presented for individual *Carcharhinus melanopterus* in good condition (**a**) and moribund (**b**). Oxygen uptake rates (*Ṁ*O_2_) were measured for 3 h after sharks were caught in gill-nets. Recovery time was extrapolated by fitting *Ṁ*O_2_ with an exponential decay function. The upper 95% confidence interval limit of minimum *Ṁ*O_2_ (horizontal line) was estimated from captive *C. melanopterus*, and the intersection of these two lines represent an individual’s extrapolated recovery time. The data in the lower panel are not fit with a recovery curve, because this individual exhibited aerobic failure when *Ṁ*O_2_ dropped below its estimated “recovered” value.

Mean *Ṁ*O_2Max_ was 322.91 ± 72.93 mg O_2_ kg^−1^ h^−1^, and EPOC was 703.72 ± 361.53 mg O_2_ kg^−1^ at 30.06 ± 1.28°C (Fig. 5a). From laboratory measurement for *C. melanopterus*, *Ṁ*O_2Min_ was 100.92 ± 11.30 mg O_2_ kg^−1^ h^−1^ at 29.66 ± 0.69°C, and estimated aerobic scope (AS = *Ṁ*O_2Max_ – *Ṁ*O_2Min_) was 221.98 mg O_2_ kg^−1^ h^−1^. No shark had recovery curves that intersected the upper 95% CI limit of *Ṁ*O_2Min_ (110.38 mg O_2_ kg^−1^ h^−1^) in under 3 h, and extrapolated recovery times ranged from 3.1 to 19.8 h (8.42 ± 5.78 h). None of the oxygen uptake parameters varied with temperature, mass, or their interaction (Supplementary Table S1). Lastly, only three *N. acutidens* were brought to the CRIOBE to generate baseline values for this species, where *Ṁ*O_2Min_ was determined to be 139.95 ± 12.07 mg O_2_ kg^−1^ h^−1^.

### Observed mortality

Immediate mortality was 0% for both species, but delayed mortality was observed for both *C. melanopterus* and *N. acutidens*. A single *C. melanopterus*, which was caught at 32.33°C, was moribund upon release from its field respirometry chamber. Oxygen uptake data suggest that this animal experienced aerobic failure at ~1.5 h following the exhaustive challenge (Fig. 5b). Including this animal, delayed mortality for *C. melanopterus* was 5.9% (1/17). The body of one *N. acutidens* was recovered the day after release from a recovery bag, suggesting that delayed mortality for this species was 25.0% (1/4); this animal was caught at 29.75°C.

## Discussion

Neonatal *C. melanopterus* and *N. acutidens* were still stressed 3 h after facing an exhaustive challenge. Values for blood glucose, lactate and pH taken 3 h after the exhaustive challenge deviated from baseline values for both species (except blood glucose concentrations in *N. acutidens*). These physiological responses are characteristic of the elasmobranch secondary stress response (Skomal and Mandelman, 2012; Wilson *et al.*, 2014). Vigorous attempts by sharks to escape fishing gear are generally supported by anaerobic metabolic pathways that are partially characterized by increases in blood glucose and lactate concentrations and a resultant drop in blood pH (Guida *et al.*, 2016; Gallagher *et al.*, 2017; Bouyoucos *et al.*, 2017b). Furthermore, sharks entangled in gill nets may not be able to ventilate, thereby driving further declines in blood pH by restricting carbon dioxide offloading, and relying on anaerobic metabolic pathways while oxygen uptake is impeded (Dapp *et al.*, 2016). Even if a shark that is restrained in a net can actively ventilate, for example via buccal pumping, this strategy could be a far less efficient method for gas exchange and may actually exacerbate the stress response (Parsons and Carlson, 1998; Brooks *et al.*, 2011). Many shark species also lack mechanisms to modulate haematological parameters related to improving oxygen delivery during a stress response (Brill and Lai, 2015). Previous studies have also documented that sharks facing brief exhaustive challenges can take over 3 h to recover (Brooks *et al.*, 2011, 2012). While we documented neonatal sharks experiencing various aspects of the stress response, it was beyond the scope of this study to determine exactly how detrimental the levels of stress experienced were (i.e. changes in recovery times or risk of experiencing mortality). Interestingly, recapture rates for both species have been relatively high within a given parturition season (~15–30%), but low recapture rates from over five years of annual surveys around Moorea suggest that natural mortality (e.g. starvation or predation) is quite high among these populations (S.P. unpublished results). Size classes between neonates and adults are notably absent from gill-net and hook-and-line surveys; although, variable habitat use or size-selective gears may appear to suggest high juvenile mortality in the absence of natural mortality rate estimates for this population (Mourier *et al.*, 2013b). Around Moorea, exhaustive challenges are expected in the form of artisanal and recreational fisheries bycatch and predator-prey interactions (Chin *et al.*, 2015; Mourier *et al.*, 2017; Thiault *et al.*, 2017). Although French Polynesia is a shark sanctuary, implementing and enforcing management strategies to mitigate fishing pressure during parturition months could reduce neonatal sharks’ chance of facing exhaustive challenges (i.e. fishing capture).

Evidence of an effect of temperature on the physiological status of *C. melanopterus* and *N. acutidens* was equivocal over a narrow, albeit ecologically relevant temperature range. Blood glucose concentrations doubled, on average, over a 3.0°C range for *C. melanopterus* (27.9–30.9°C) and [Hb] increased with temperature by ~23% over a 1.4°C range (29.5–30.9°C) in *N. acutidens*. Some markers of physiological status may respond to changing environmental temperatures for elasmobranchs because of temperature’s influence on the metabolic rates of ectothermic organisms (Hoffmayer *et al.*, 2012; Guida *et al.*, 2016). Conversely, temperature-associated changes in blood glucose concentrations of *C. melanopterus* could simply reflect increased activity levels of sharks in warmer water, as opposed to a temperature-mediated metabolic response (Whitney *et al.*, 2016; Payne *et al.*, 2016, 2018). Increases in [Hb] of *N. acutidens* with increasing temperature may be a compensatory mechanism as Hb–O_2_ affinity decreases (Bernal *et al.*, 2018). Alternatively, the apparent correlation between [Hb] and temperature may have been spurious, as changes in [Hb] ultimately did not result in variation in MCHC or Hct. However, *N. acutidens* in warmer waters may have had smaller red blood cells (RBCs) or immature RBCs in greater circulation but with similar [Hb] to sharks at cooler temperatures that would appear as an increase in [Hb] without affecting other haematological variables. No other physiological or oxygen uptake parameters that were measured displayed variations with temperature. Metabolic compensation, where an organism maintains consistent *Ṁ*O_2_ with temperature acclimation, has not been documented for elasmobranchs (Tullis and Baillie, 2005). It is likely, however, that, even for seasonally-acclimated elasmobranchs, variations in temperature exceeding 3.0°C may be necessary to elicit an observable response (Carlson and Parsons, 1999; Neer *et al.*, 2006). Moorea’s neonatal shark populations face summer temperatures that average 30°C during parturition months, daily variations of up to 8°C, and extreme temperatures ranging 26–36°C (J.L.R. unpublished results). For juvenile sharks, facing an exhaustive challenge in shallow coastal waters when temperatures are high can be lethal (Danylchuk *et al.*, 2014). The only *C. melanopterus* to die in this study was, coincidentally, captured at >32°C, but we could not confirm whether this single mortality was related to temperature. Both *C. melanopterus* and *N. acutidens* exhibit some degree of philopatry to natal areas around Moorea and elsewhere, such that extreme temperature events in these potential nursery areas could put neonates at risk of mortality after facing exhaustive challenges (Mourier and Planes, 2013; Mourier *et al.*, 2013b; Oh *et al.*, 2017a). Without controlled studies to investigate the effect of temperature on reef sharks’ resilience to stress, it is unclear whether thermal stressors like ocean warming brought on by climate change could be problematic for neonatal sharks in tropical nearshore habitats.

Physiological status before and after the exhaustive challenge was species-specific. Notably, *N. acutidens* exhibited a larger difference in blood pH relative to baseline values, did not exhibit variation in blood glucose concentrations across the samples, and had high baseline lactate concentrations compared to *C. melanopterus*. Overall trends in blood lactate concentrations, blood pH and haematological parameters, however, were similar for both species and consistent with what has been reported for other elasmobranchs (Lowe *et al.*, 1995; Richards *et al.*, 2003; Brill *et al.*, 2008). The higher *Ṁ*O_2Min_ observed for *N. acutidens* could explain the larger drop in blood pH (ΔpH = 0.44) following the exhaustive challenge relative to *C. melanopterus* (ΔpH = 0.28). It is hypothesised that the magnitude and severity of a stress response is related to *Ṁ*O_2_ for elasmobranchs (Skomal and Mandelman, 2012). While it was not possible to calculate AS for both species, *Carcharhinus melanopterus* are generally regarded as stronger aerobic swimmers than *N. acutidens*, which are less active and known to rest (Baldwin and Wells, 1990; Wells *et al.*, 1992). High blood glucose concentrations and resting blood lactate concentrations in *N. acutidens* could be a result of this species recruiting anaerobic metabolism to support bouts of swimming that are interspersed with periods of resting (Piiper *et al.*, 1977). Blood-oxygen transport properties ([Hb], Hct, MCHC) were not affected by exercise, and were similar between the two species, as has been previously reported (Wells and Baldwin, 1990; Wells *et al.*, 1992). In addition, juvenile *C. melanopterus* and *N. acutidens* from Heron Island (on the Great Barrier Reef) were reported to exhibit similarly pH-insensitive haemoglobins, suggesting that Hb–O_2_ affinity and oxygen transport are not greatly affected by an acidosis for these species (Baldwin and Wells, 1990; Wells *et al.*, 1992). Taken together, these data suggest that each species has a unique physiological response to stress in relation to their behaviour and aerobic capacity, where *N. acutidens* may have experienced a more intense stress response owing to a potential greater reliance on anaerobic metabolism to support activity. It would be informative to characterize the full physiological response from initiation to resolution (i.e. recovery or mortality) to determine how differently these two species respond to capture.

There was no observable immediate mortality for *C. melanopterus* and *N. acutidens*. However, delayed mortality rates were higher for *N. acutidens* (25%) than *C. melanopterus* (5.9%), although this study’s experimental design precluded quantification of robust mortality rates for either species. Adult *C. melanopterus* around Moorea appear to be quite resilient to hook-and-line capture (Mourier *et al.*, 2017), and neonate and juvenile *C. melanopterus* and *N. acutidens* both exhibited near 0% mortality following gill-net and hook-and-line capture in the Mangrove Bay Sanctuary Zone on Ningaloo Reef, Australia (Oh *et al.*, 2017a). In contrast, however, another study out of Western Australia reported that, when facing unspecified capture durations in gill-nets, juvenile and adult *C. melanopterus* were more susceptible to immediate mortality than *N. acutidens* (Dapp *et al.*, 2017). Local adaptation to environmental conditions at the population level may influence these contrasting trends (Eliason *et al.*, 2011; Di Santo, 2016); although, temperature data were not reported from the Western Australia study (Dapp *et al.*, 2017). The model generated by Dapp *et al.* (2017) to estimate immediate mortality of *C. melanopterus* would have predicted 100% mortality for sharks in our study using only total length as a predictor. It is possible that differences in the duration of capture led this study to conclude that immediate mortality was 0%, whereas difficulty in identifying capture events by Dapp *et al.* (2017) could have allowed for sufficiently long capture durations and more realistic immediate mortality estimates. Alternatively, stress resulting from this study’s shorter capture durations may simply not have been fatal (Oh *et al.*, 2017a). Differences in mortality estimates for *N. acutidens* may have been related to size; although, sizes of *N. acutidens* were not reported by Dapp *et al.* (2017). It, therefore, seems likely that these apparently contrasting findings resulted from differences in the nature and duration of the stressor (e.g. capture duration, supplementing air exposure, local environmental conditions, etc.).

This study’s exhaustive challenge was associated with a large energetic cost and long recovery for *C. melanopterus*. The mean estimated EPOC was 703.72 mg O_2_ kg^−1^, and recovery was estimated to take 8.42 h. Comparatively, chasing juvenile lemon sharks (*N. brevirostris*) to exhaustion without air exposure resulted in an EPOC of 154.10 mg O_2_ kg^−1^ and 5.40 h of recovery at 30°C (Bouyoucos *et al.*, 2017b). Gill-net capture and air exposure may result in a larger EPOC than exhaustive chasing because oxygen uptake is impeded, such that recovery cannot begin until oxygen uptake is resumed; a chased fish in water can still meet some of its energy demand aerobically and even begin to recover. The EPOC estimated for *C. melanopterus* is much larger than measured for other elasmobranchs (Brett and Blackburn, 1978; Bouyoucos *et al.*, 2017a, b), but it is similar to values reported for a tropical coral reef fish (*Pomacentrus amboinensis*) at comparable temperatures (28–29°C) (Killen *et al.*, 2014). However, recovery times for *P. amboinensis* were under one hour, which likely relates to this species’ impressive AS that is almost ten times that of *C. melanopterus* (Killen *et al.*, 2014). Assuming that *Ṁ*O_2_ scales with swimming speeds similarly among carcharhinid sharks (Carlson *et al.*, 2004), a routine swimming *Ṁ*O_2_ of 195.87 mg O_2_ kg^−1^ h^−1^ can be estimated for *C. melanopterus* using a power-performance slope of 0.36, a routine swimming speed of 0.80 body lengths s^−1^ for captive *C. melanopterus*, and this study’s estimate of *Ṁ*O_2Min_ (Webb and Keyes, 1982; Bushnell *et al.*, 1989). Applying an oxygen equivalent of 14.14 J mg O_2_^−1^, *C. melanopterus* would have a daily metabolic rate of 66.47 kJ kg^−1^ d^−1^ for swimming alone, and an EPOC of 9.95 kJ kg^−1^ from an exhaustive challenge would increase daily energy expenditure for swimming by 14.9% (Elliott and Davison, 1975). Around Moorea, neonatal *C. melanopterus* (and *N. acutidens*) must quickly transition from relying on endogenous fuel stores to energy acquired through hunting (Matich *et al.*, 2015). Energetically costly one-off exhaustive challenges, like incidental capture, could precede starvation in neonatal sharks, especially for populations with high natural mortality.

In conclusion, within a narrow range of temperatures, neonatal *C. melanopterus* and *N. acutidens* are resilient to brief durations of gill-net capture. However, we are unaware of these species’ physiological resilience to longer durations of capture with different gear types, to longer periods of air exposure, or at temperatures beyond 28–31°C. As such, artisanal and recreational fisheries bycatch mortality could still pose a threat to Moorea’s neonate and juvenile shark populations. Indeed, longer gill-net capture durations could be fatal, at least for *C. melanopterus* (Dapp *et al.*, 2017). Moving forward, studies are needed to define environmental conditions that limit physiological performance and to fully characterize recovery following a challenge. Furthermore, defining changes in routine energy requirements and reserves of neonates exposed to stressors in relation to the quality and availability of shelter and prey will be important for estimating sharks’ likelihood of facing predation and starvation, respectively. Together, these data have the potential to improve our understanding of how anthropogenic and environmental stressors affect the survivorship of neonate and juvenile reef sharks in important habitats like shark nursery areas. Understanding the vulnerability of shark populations to manageable stressors, like fishing pressure, is an important step toward improving the efficacy of MPAs as conservation tools for sharks, globally.

## Supplementary Material

Supplementary Data
