# Supplementary material for: Dead tired: evaluating the physiological status and survival of neonatal reef sharks under stress
Source: Conserv Physiol. 2018 Sep 18;6(1):coy053. doi: 10.1093/conphys/coy053 (PMC6142904; doi:10.1093/conphys/coy053)
Supplement: Supplementary Data [file coy053_cp_bouyoucos_et_al_supplementary_tables.docx]

# Supplementary Tables

**Supplementary Table S1:** Linear model output for physiological parameters (response) fit with treatment, mass, and temperature as factors for blacktip reef sharks (*Carcharhinus melanopterus*). Oxygen uptake parameters (response) were fit with temperature and mass as covariates. Abbreviations: excess post-exercise oxygen consumption (EPOC), haematocrit (Hct), haemoglobin concentration (Hb), maximum oxygen uptake rate ($\dot{M}$O_2Max_), mean cell haemoglobin concentration (MCHC), minimum oxygen uptake rate ($\dot{M}$O_2Min_).

| **Response** | **Factor** | **D.F.** | ***F*-value** | ***p*-value** |
| --- | --- | --- | --- | --- |
| **Glucose** | **Treatment** | **3, 15** | **14.75** | **< 0.001** |
|  | **Temperature** | **1, 15** | **6.77** | **0.020** |
|  | Mass | 1, 15 | 2.77 | 0.117 |
|  | Treatment × Temperature | 3, 15 | 1.16 | 0.359 |
|  | Treatment × Mass | 3, 15 | 2.06 | 0.148 |
|  | Temperature × Mass | 1, 15 | 0.67 | 0.424 |
|  | Treatment × Temperature × Mass | 3, 15 | 1.82 | 0.186 |
| **Lactate** | **Treatment** | **3, 15** | **340.91** | **< 0.001** |
|  | Temperature | 1, 15 | 1.39 | 0.256 |
|  | Mass | 1, 15 | 0.94 | 0.348 |
|  | Treatment × Temperature | 3, 15 | 1.86 | 0.180 |
|  | Treatment × Mass | 3, 15 | 0.44 | 0.729 |
|  | Temperature × Mass | 1, 15 | 0.00 | 0.985 |
|  | Treatment × Temperature × Mass | 3, 15 | 1.21 | 0.340 |
| **pH** | **Treatment** | **3, 15** | **11.58** | **< 0.001** |
|  | Temperature | 1, 15 | 0.03 | 0.865 |
|  | Mass | 1, 15 | 0.27 | 0.609 |
|  | Treatment × Temperature | 3, 15 | 0.73 | 0.551 |
|  | Treatment × Mass | 3, 15 | 0.07 | 0.977 |
|  | Temperature × Mass | 1, 15 | 0.01 | 0.923 |
|  | Treatment × Temperature × Mass | 3, 15 | 0.76 | 0.533 |
| Hb | Treatment | 3, 15 | 0.17 | 0.914 |
|  | Temperature | 1, 15 | 0.27 | 0.613 |
|  | Mass | 1, 15 | 0.40 | 0.127 |
|  | Treatment × Temperature | 3, 15 | 0.37 | 0.771 |
|  | Treatment × Mass | 3, 15 | 0.87 | 0.480 |
|  | Temperature × Mass | 1, 15 | 0.04 | 0.846 |
|  | Treatment × Temperature × Mass | 3, 15 | 1.32 | 0.306 |
| Hct | Treatment | 3, 15 | 0.14 | 0.934 |
|  | Temperature | 1, 15 | 0.34 | 0.570 |
|  | Mass | 1, 15 | 1.63 | 0.221 |
|  | Treatment × Temperature | 3, 15 | 0.78 | 0.526 |
|  | Treatment × Mass | 3, 15 | 0.41 | 0.764 |
|  | Temperature × Mass | 1, 15 | 0.13 | 0.719 |
|  | Treatment × Temperature × Mass | 3, 15 | 0.54 | 0.662 |
| MCHC | Treatment | 3, 15 | 0.76 | 0.536 |
|  | Temperature | 1, 15 | 0.04 | 0.855 |
|  | Mass | 1, 15 | 0.03 | 0.873 |
|  | Treatment × Temperature | 3, 15 | 1.56 | 0.240 |
|  | Treatment × Mass | 3, 15 | 0.53 | 0.669 |
|  | Temperature × Mass | 1, 15 | 0.00 | 0.941 |
|  | Treatment × Temperature × Mass | 3, 15 | 0.43 | 0.736 |
| $\dot{M}$O_2Max_ | Temperature | 1, 4 | 0.527 | 0.508 |
|  | Mass | 1, 4 | 0.018 | 0.901 |
|  | Temperature × Mass | 1, 4 | 1.786 | 0.252 |
| $\dot{M}$O_2Min_ | Temperature | 1, 4 | 0.053 | 0.829 |
|  | Mass | 1, 4 | 0.160 | 0.710 |
|  | Temperature × Mass | 1, 4 | 2.446 | 0.191 |
| EPOC | Temperature | 1, 4 | 0.654 | 0.464 |
|  | Mass | 1, 4 | 0.483 | 0.525 |
|  | Temperature × Mass | 1, 4 | 5.014 | 0.088 |
| Recovery Time | Temperature | 1, 4 | 1.192 | 0.336 |
|  | Mass | 1, 4 | 0.009 | 0.931 |
|  | Temperature × Mass | 1, 4 | 2.949 | 0.161 |

**Supplementary Table S2:** Linear model output for physiological parameters (response) fit with treatment, temperature and mass as factors for sicklefin lemon sharks (*Negaprion acutidens*). Abbreviations: haematocrit (Hct), haemoglobin concentration (Hb), mean cell haemoglobin concentration (MCHC).

| **Response** | **Factor** | **D.F.** | ***F*-value** | ***p*-value** |
| --- | --- | --- | --- | --- |
| Glucose | Treatment | 2, 8 | 1.551 | 0.270 |
|  | Temperature | 1, 8 | 0.305 | 0.596 |
|  | Mass | 1, 8 | 0.504 | 0.498 |
| **Lactate** | **Treatment** | **2, 8** | **50.211** | **< 0.001** |
|  | Temperature | 1, 8 | 1.866 | 0.209 |
|  | Mass | 1, 8 | 0.135 | 0.723 |
| **pH** | **Treatment** | **2, 8** | **6.593** | **0.018** |
|  | Temperature | 1, 8 | 0.002 | 0.968 |
|  | Mass | 1, 8 | 0.631 | 0.449 |
| **Hb** | Treatment | 2, 8 | 1.018 | 0.404 |
|  | **Temperature** | **1, 8** | **6.318** | **0.036** |
|  | Mass | 1, 8 | 0.167 | 0.693 |
| Hct | Treatment | 2, 8 | 0.795 | 0.484 |
|  | Temperature | 1, 8 | 0.157 | 0.703 |
|  | Mass | 1, 8 | 0.070 | 0.797 |
| MCHC | Treatment | 2, 8 | 0.372 | 0.701 |
|  | Temperature | 1, 8 | 2.780 | 0.134 |
|  | Mass | 1, 8 | 0.052 | 0.826 |
